# Supplementary material for: Mechanism of client loading from BiP to Grp94 and its disruption by select inhibitors
Source: Nat Commun. 2025 Apr 15;16:3575. doi: 10.1038/s41467-025-58658-w (PMC12000397; doi:10.1038/s41467-025-58658-w)
Supplement: Supplementary file 1 — Supplementary Information [file 41467_2025_58658_MOESM1_ESM.pdf]

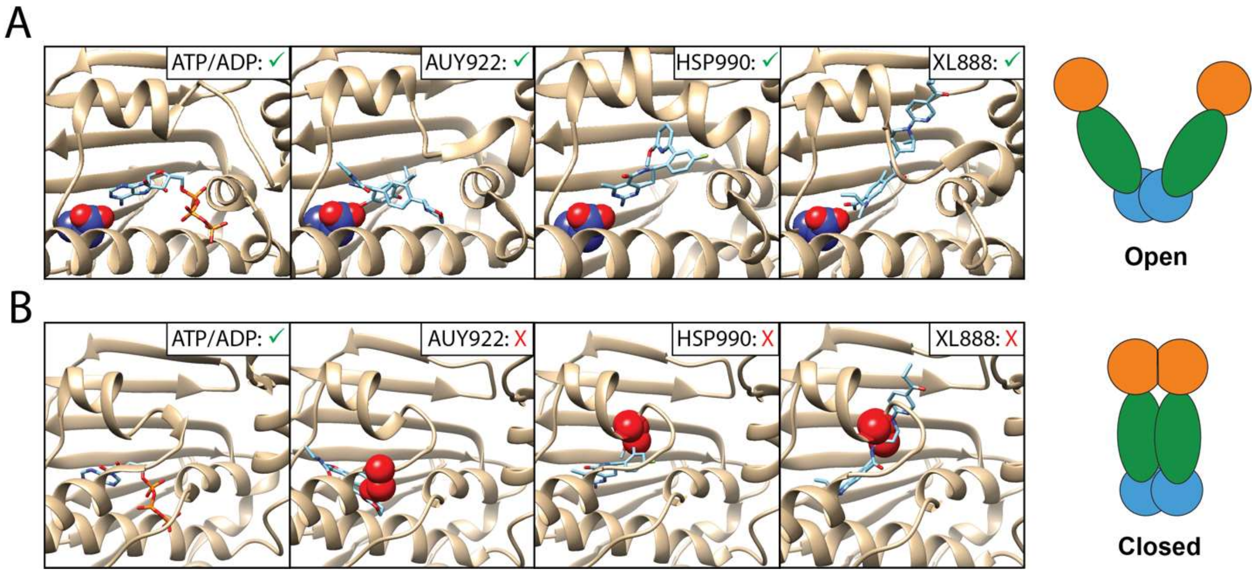

Supplementary Figure 1. A. Structures of ATP (PDB: 3T0Z), AUY922 (PDB: 6LTI), HSP990 (PDB: 4U93), and XL888 (PDB: 4AWO) bound to the Hsp90α NTD. Asp93, which forms key contacts with both nucleotide and inhibitors, is shown in dark blue. **B.** Superposition of structures containing AUY922, HSP990, and XL888 onto the Hsp90α closed structure (PDB: 5FWK). Hsp90 structures are shown in tan, nucleotides/inhibitors shown in light blue, and residues that clash with inhibitors are shown in red.

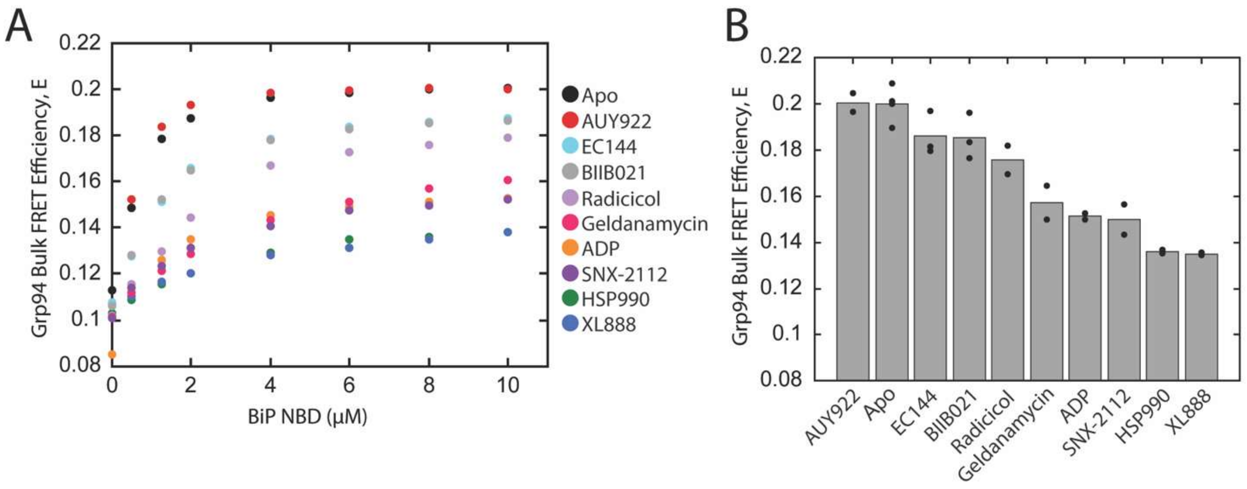

Supplementary Figure 2. A. Grp94 bulk FRET efficiency at varying concentrations of BiP NBD with no inhibitor (black), 1mM ADP (orange), or 50μM inhibitor. Points are averages from independent replicate measurements (apo: n=4; AUY992, HSP990, SNX-2112, XL888, Radicicol, Geldanamycin, ADP: n=2; EC144, BIIB021: n=3). **B.** Grp94 bulk FRET efficiency at 8μM BiP NBD. Data points are from independent replicate experiments. Source data are provided as a Source Data file.

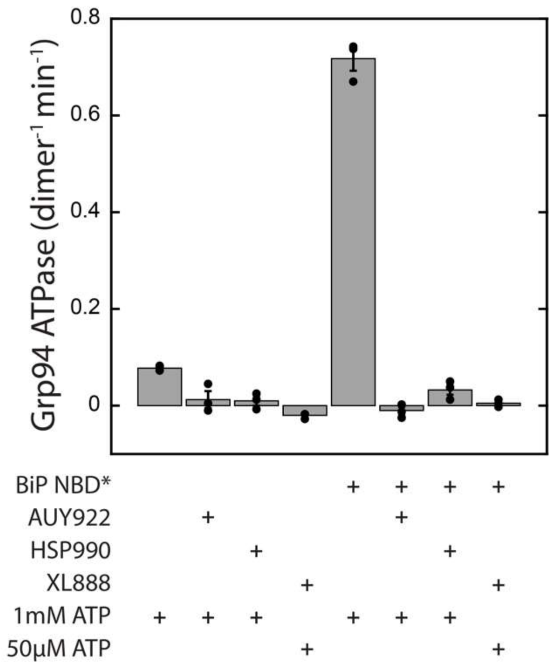

Supplementary Figure 3. Grp94 ATPase in the presence and absence of 8μM BiP NBD* and 50μM inhibitors (AUY922, HSP990, XL888). Error bars are the SEM for three independent replicate measurements. Source data are provided as a Source Data file.

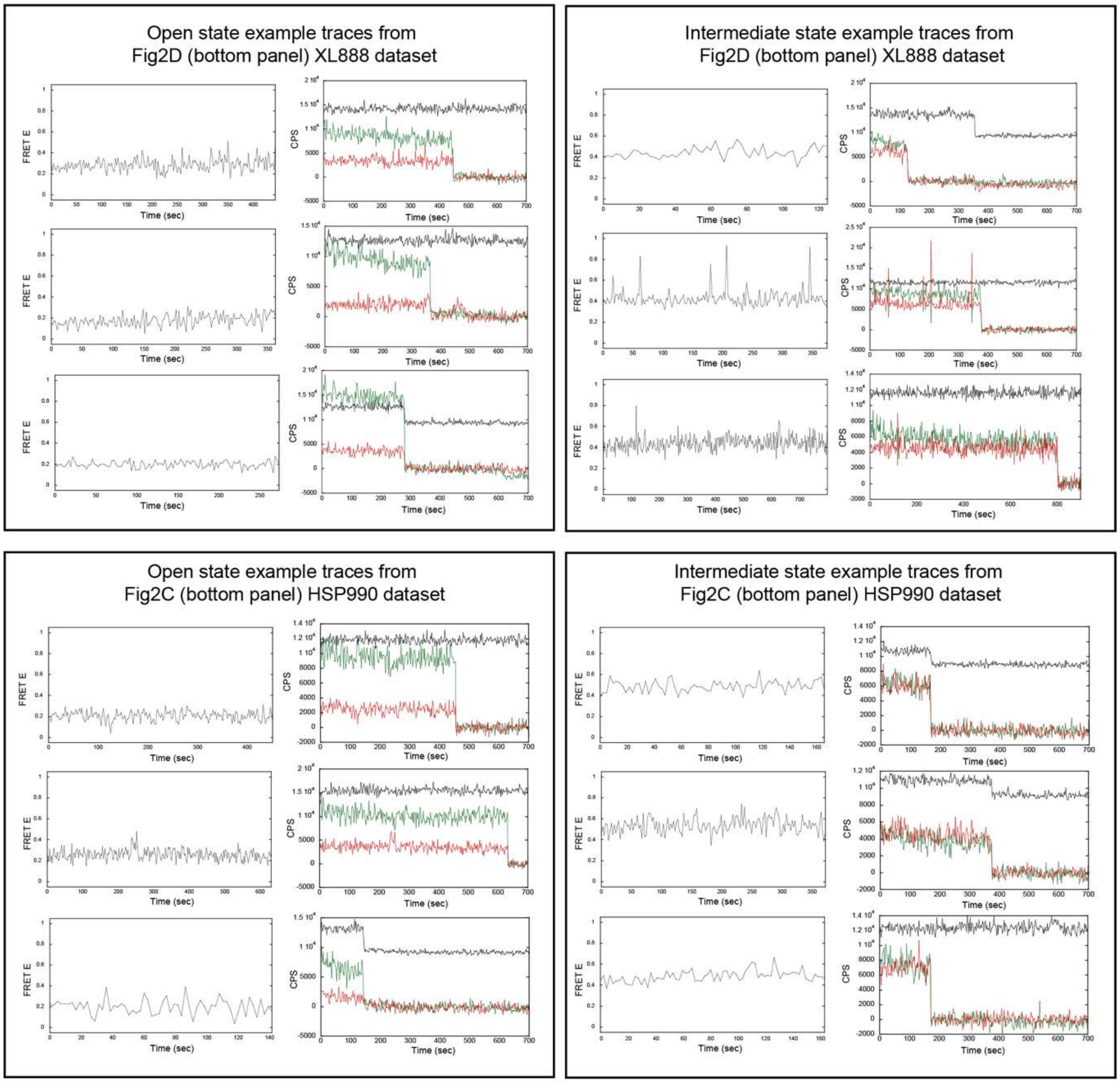

Supplementary Figure 4: Example FRET traces (left) and corresponding donor, acceptor, and direct excitation traces (right) of individual Grp94 dimers corresponding to the open conformation or intermediate FRET conformation. These data correspond to histograms in **Figure 2D**, in conditions with 8µM BiP NBD and 50µM XL888 or HSP990. Donor fluorescence is shown in green, acceptor fluorescence is shown in red, and direct acceptor excitation fluorescence is shown in black.

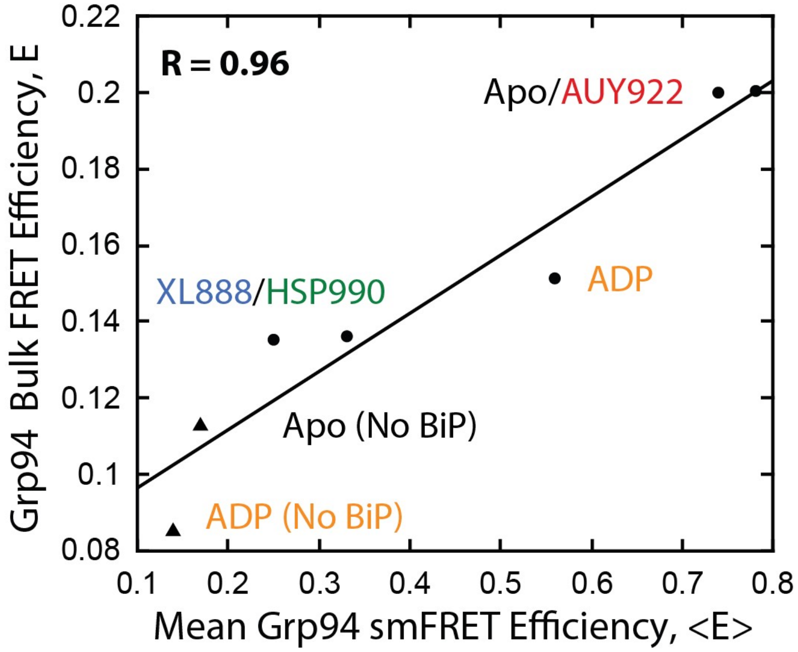

Supplementary Figure 5. Relationship between average Grp94 FRET efficiencies (<E>) measured by smFRET (from **Figure 2A-D**) and average bulk FRET efficiencies (from **Supplementary Figure 2A**) with (circles) and without (triangles) 8μM BiP NBD. Solid line is a linear fit with correlation coefficient (R) shown. Bulk FRET efficiencies are lower than smFRET efficiencies due to incomplete fluorophore labelling and signal contributions in bulk FRET from Grp94 homodimers with either two acceptor or two donor fluorophores. Source data are provided as a Source Data file.

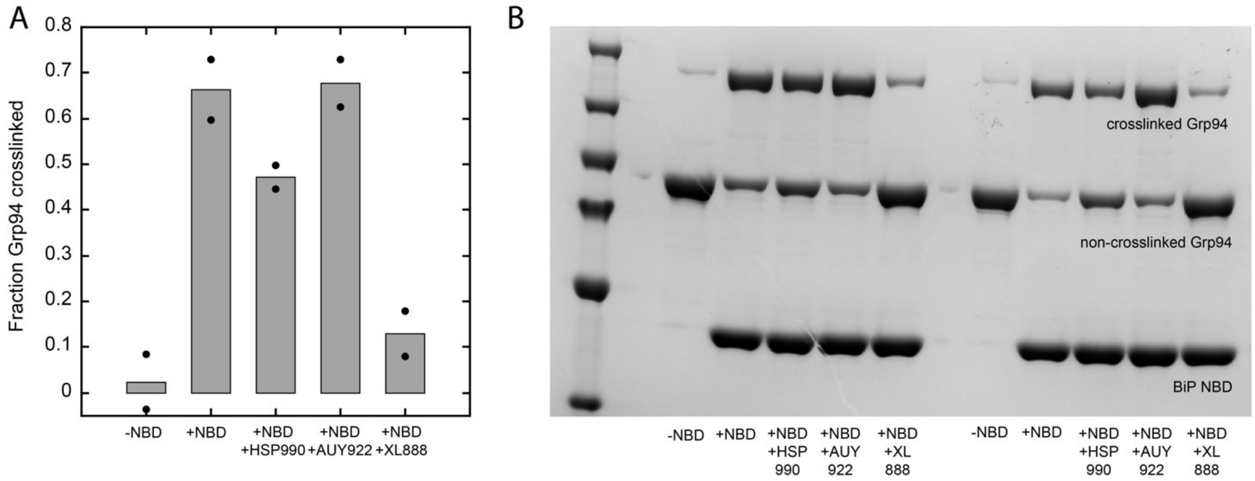

Supplementary Figure 6: A. Quantification of M86C Grp94 crosslinking as measured by integrating bands on a non-reducing SDS PAGE. **B.** Non-reducing SDS PAGE gel of M86C crosslinking experiments. Unless otherwise indicated, samples contain 5µM BiP NBD; in samples with inhibitor the concentration is 50µM. Crosslinking was quenched at 2 hours. Bar represents the average from two independent experiments. Source data are provided as a Source Data file.

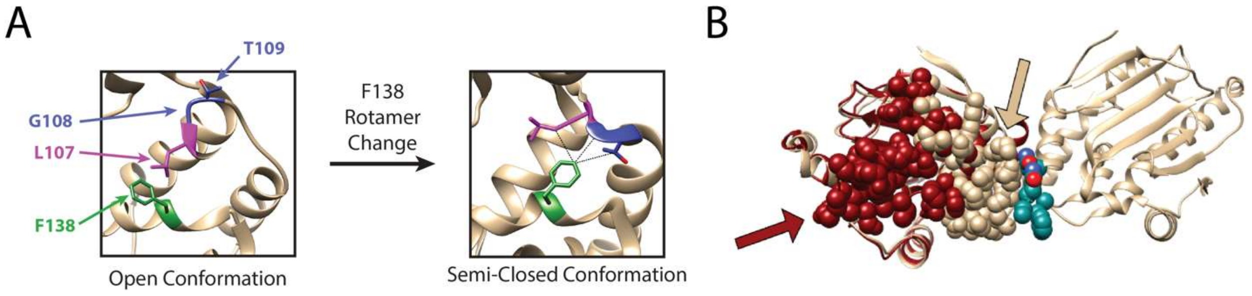

Supplementary Figure 7. A. $Hsp90_{F138}$ (green) rotamer change between the Hsp90α open (left, PDB: 5J2V) and semi-closed (right, PDB: 7KW7) conformations. Residues involved in the hydrophobic cluster are labeled. $Hsp90_{L107}$ is shown in magenta and $Hsp90_{G108}$ and $Hsp90_{T109}$ are shown in blue on the structures. The hydrophobic cluster is indicated by black dashed lines between the residues (right). **B.** Overlay of NTDs from Hsp90 semi-closed conformation (tan, PDB: 7KW7) and Hsp90 closed state (red, PDB: 5FWK). The semi-closed NTD dimer interface is shown in teal. The lid structures are shown in space filling. The tan arrow is pointing to lid conformation in the semi-closed conformation and the red arrow is pointing to the lid conformation in closed state.

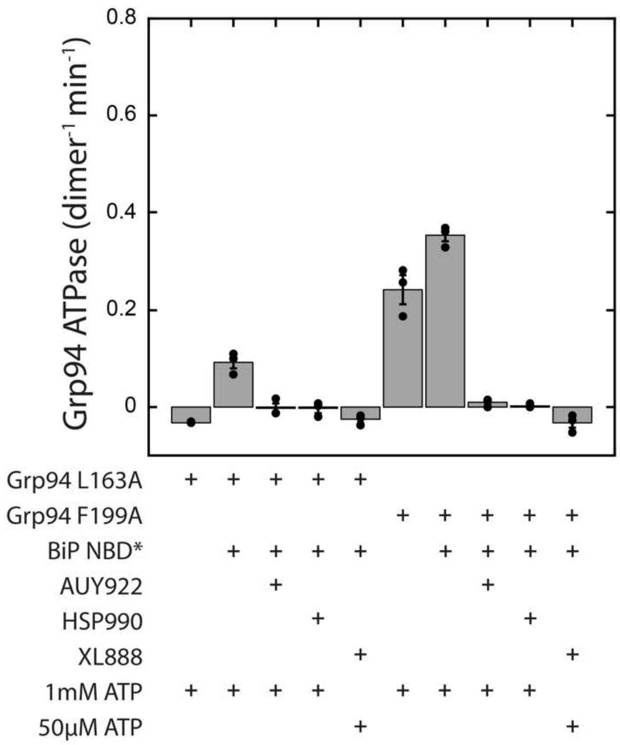

Supplementary Figure 8. Grp94$_{L163A}$ and Grp94$_{F199A}$ ATPase in the presence and absence of 8µM BiP NBD* and 50µM inhibitors (AUY922, HSP990, XL888). Error bars are the SEM for three independent replicate measurements. Source data are provided as a Source Data file.

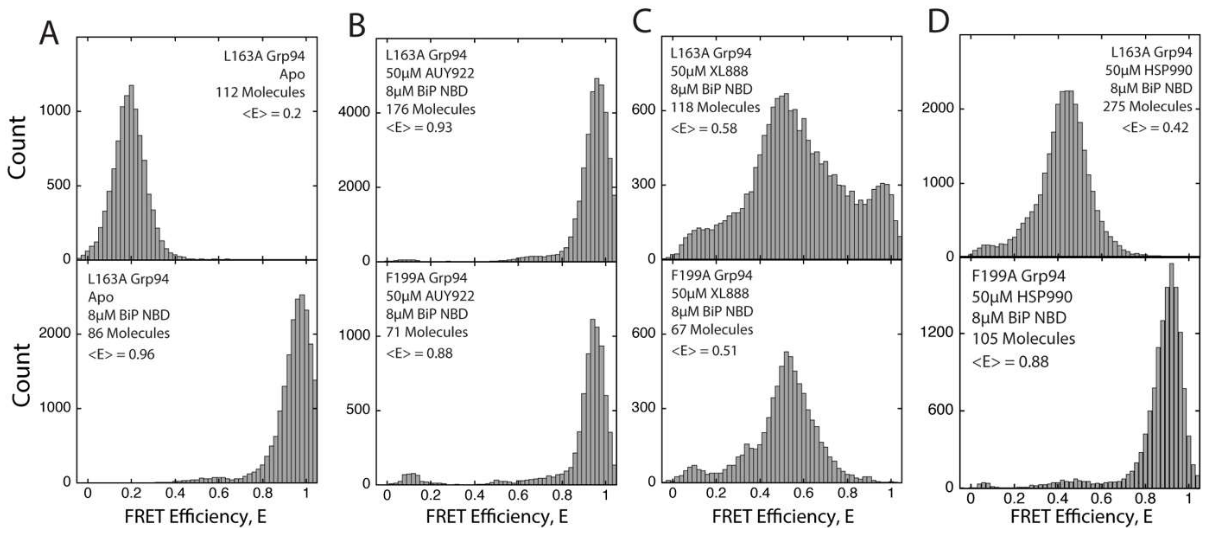

Supplementary Figure 9: $Grp94_{L163A}$ and $Grp94_{F199A}$ smFRET efficiency histograms. Results are shown for measurements in the absence of any ligand (Apo, **A**), and in the presence of 50µM AUY922 (**B**), 50µM XL888 (**C**), 50µM HSP990 (**D**). <E> is the average FRET efficiency for the entire histogram. See Methods for experimental details. Source data are provided as a Source Data file.

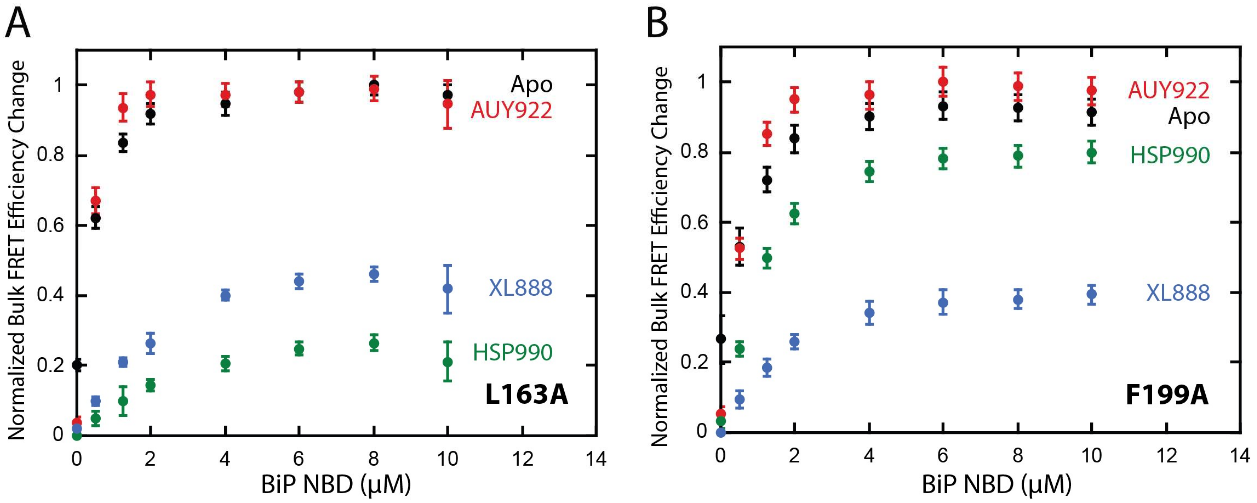

Supplementary Figure 10. Grp94$_{L163A}$ (**A**) and Grp94$_{F199A}$ (**B**) normalized bulk FRET efficiency change at varying concentrations of BiP NBD with no inhibitor (black) or 50μM AUY922 (red), XL888 (blue), or HSP990 (green). Data at 8μM BiP NBD is shown in **Figure 3A**. Error bars are the propagated SEM from three independent replicate measurements. Source data are provided as a Source Data file.

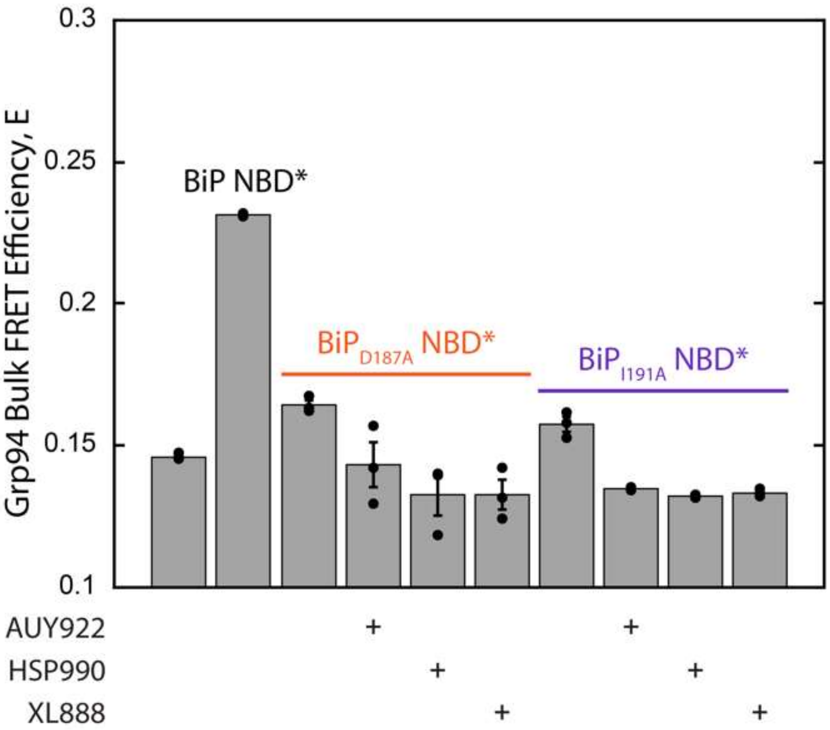

Supplementary Figure 11: Grp94 bulk FRET efficiency with 8µM of different BiP NBD constructs in the presence and absence of 50µM inhibitors. Error bars are the SEM from three independent replicate measurements. Experimental conditions are the same as in **Figure 4B** except with 2% DMSO. Source data are provided as a Source Data file.

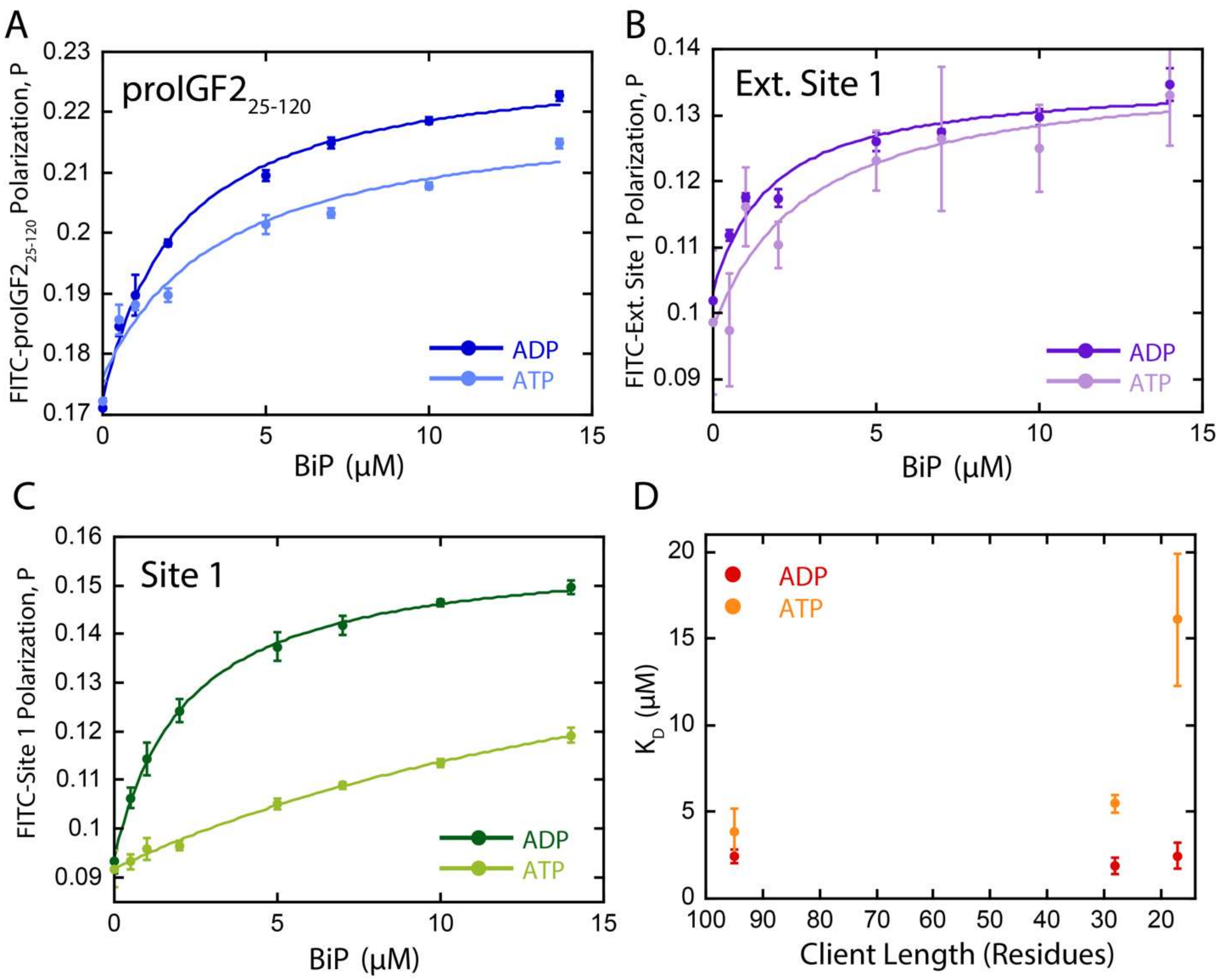

Supplementary Figure 12A-C. FP BiP binding assay with FITC-labeled clients. Solid lines are fit to a single site binding model. **A.** BiP affinities for proIGF2$_{25\text{-}120}$ are 2.5±0.4μM and 3.8±1.4μM under ADP and ATP conditions respectively. **B.** BiP affinities for Ext. Site 1 (proIGF2$_{92\text{-}120}$) are 1.9±0.5μM and 5.5±0.5μM under ADP and ATP conditions respectively. **C.** BiP affinities for site 1 (proIGF2$_{103\text{-}120}$) are 2.5±0.7μM and 16±3.8μM under ADP and ATP conditions respectively. **D.** BiP affinities for clients of decreasing size under ADP and ATP conditions. Client constructs include: proIGF2$_{25\text{-}120}$ (95 residues), ext. site 1 (28 residues), and site 1 (17 residues). Error bars are the SEM of three independent replicate measurements. Source data are provided as a Source Data file.

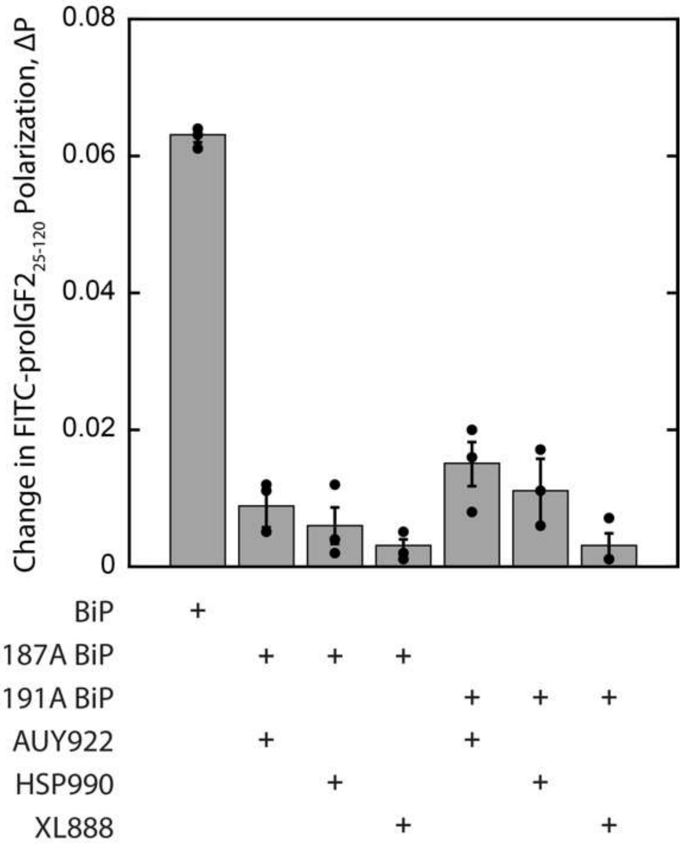

Supplementary Figure 13: Change in FP (ΔP) of FITC-labeled proIGF2$_{25-120}$ with 5µM BiP, BiP$_{D187A}$, or BiP$_{I191A}$, upon addition of 5µM Grp94 in the presence of 50µM inhibitors. All conditions include 1mM ADP. Error bars are the SEM of three independent replicate measurements. Source data are provided as a Source Data file.

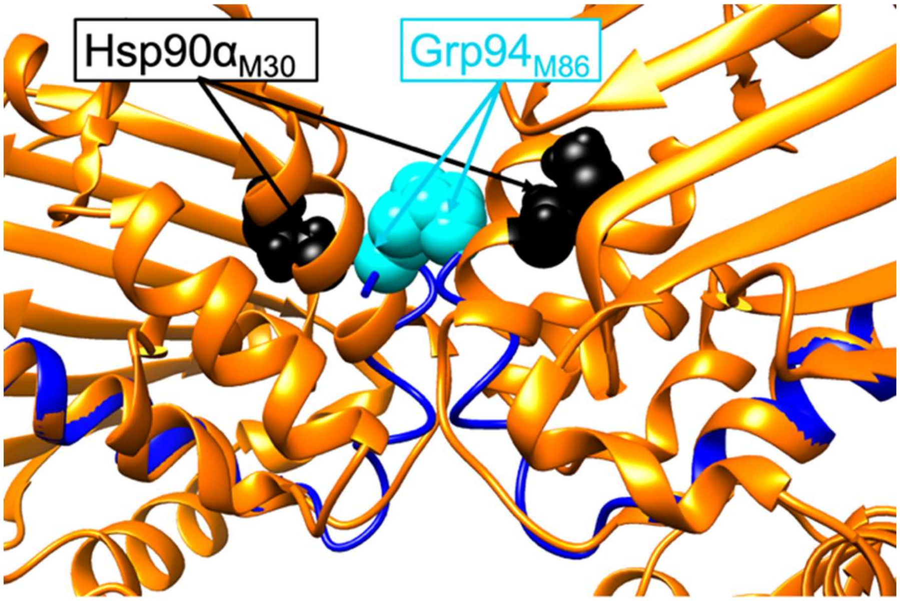

Supplementary Figure 14. Hsp90 semi-closed state NTD (PDB: 7KW7, orange) with superimposed partially folded α helices (residues 85-120) from Grp94 open state (PDB: 2O1V, blue). The superposition was performed with MatchMaker on UCSF Chimera[1]. The residues that can form a disulfide bond with cysteine mutations on Grp94 are shown in cyan (Grp94$_{M86}$, with a distance between β carbons of 3Å). The corresponding residues on Hsp90α (Hsp90α$_{M30}$, with a distance between β carbons of 18Å) are shown in black.

	Residue	Distance (Å)
Grp94 closed state	Met86	16
Trap 1 coiled-coil state	Leu98	6
Hsp90 semi-closed state	Met30	18
Hsp90 semi-closed state with open state α helices	Met86	3

Supplementary Table 1. Distances between the β carbons of residue pairs on opposite arms in the Grp94 closed state (PDB: 5ULS), Trap 1 coiled-coil state (PDB: 5F3K), Hsp90 semi-closed state (PDB: 7KW7), and Hsp90 semi-closed state with open state α helices (from **Supplementary Figure 14**).

Supplementary References

1. Pettersen, E. F. *et al.* UCSF Chimera--a visualization system for exploratory research and analysis. *J Comput Chem* **25**, 1605–1612 (2004).
